# Clinicopathological analysis of hyalinizing clear cell carcinoma in the head and neck

**DOI:** 10.1016/j.bjorl.2025.101676

**Published:** 2025-07-29

**Authors:** Xiaoli Zhao, Donglin Ma, Yahui Li, Dongmei Yang, Yingshi Piao

**Affiliations:** Department of Pathology, Beijing Tongren Hospital, Capital Medical University, Beijing Key Laboratory of Head and Neck Molecular Diagnostic Pathology, Beijing, China

**Keywords:** Head and neck tumors, Salivary glands, Hyalinizing clear cell carcinoma, Clinical pathological features, Fluorescence in situ hybridization

## Abstract

**Objective:**

To study clinicopathological features and differential diagnosis of hyalinizing clear cell carcinoma in the head and neck.

**Methods:**

A retrospective analysis was performed patients with hyalinizing clear cell carcinoma in the head and neck, who underwent surgical resection in Beijing Tongren Hospital affiliated to Capital Medical University from January 2014 to May 2024. The clinical features of HCCC were analyzed by computed tomography and magnetic resonance imaging. Tumor sections were further characterized using hematoxylin and eosin staining, immunohistochemistry, and fluorescence in situ hybridization.

**Results:**

There were 4 male and 5 female cases in 9 patients. The age range was 28–84 years-old, the middle age was 60 years-old. 9 patients, 5 in the nasopharynx, 2 in the base of the tongue, and 2 in the oropharynx. At the cellular level, the HCCC tumors were composed of transparent and eosinophilic cells (in various ratios), which were typically arranged into nests, cords, and trabeculae, and embedded in a glassy hyaline substance. Immunohistochemical evaluation showed positivity for CK7, P63, P40, CK5/6, and negativity for S100, SMA, GFAP, and calponin. Fluorescence in situ hybridization revealed EWSR1 rearrangement in all the nine patients evaluated.

**Conclusions:**

Hyalinizing clear cell carcinoma is a rare low-grade salivary gland carcinoma that mostly occurs in the minor salivary glands. They had an indolent disease course, with few metastases. Our study shows that the above combination of analytical techniques can be used to accurately characterize and diagnose HCCC.

**Level of evidence:**

Level 1.

## Highlights

• Hyalinizing Clear Cell Carcinoma (HCCC) is a rare, low-grade malignant tumor.
• HCCC is frequent misdiagnosis.
• Combination of analytical techniques can be used to accurately diagnose HCCC.

## Introduction

Hyalinizing Clear Cell Carcinoma (HCCC) is a rare, low-grade malignant tumor, which typically develops in the minor salivary glands. Because of its characteristic pathological features, HCCC was initially labelled as a non-specific type of clear cell carcinoma in the third edition of the World Health Organization (WHO) classification of head and neck tumors.^1^ The 2016 WHO classification of salivary tumors referred to HCCC as salivary gland clear cell carcinoma,^2^ before the term HCCC was finally adopted in the fifth edition (2022) of the WHO classification of head and neck tumors.^3^ Despite these attempts to classify HCCC, our understanding of this disease is still limited, leading to its frequent misdiagnosis. The aim of the present study was to explore the clinicopathological, histological, immunophenotypic, and molecular features of HCCC in the head and neck in nine patients treated at our institution to improve our understanding of this disease.

## Methods

### Ethical justification

The study was approved by the Ethical Committee of Beijing Tongren Hospital affiliated to Capital Medical University.

### Patients and tissue samples

Data collected from patients with HCCC in the head and neck, who were treated at the Beijing Tongren Hospital affiliated to Capital Medical University from January 2014 to May 2024, were retrospectively analyzed. After evaluation by two senior pathologists, nine patients with HCCC were enrolled in the study. Patients with primary tumors in the head and neck and a confirmed diagnosis of HCCC were included, while those with a history of previous tumors or with synchronous multiple primary tumors were excluded.

The Formalin-Fixed Paraffin-Embedded (FFPE) tissue samples containing the invasive part of the tumor were obtained and subjected to Hematoxylin and Eosin (H&E) staining, Immunohistochemistry (IHC), and EWSR1 Fluorescence In Situ Hybridization (FISH). The sections were observed under a BX50 microscope (Olympus, Shinjuku City, Japan).

### Study groups and follow-up

Clinical data relating to age, sex, tumor location, and the time of recurrence, metastasis, or death were recorded. The enrolled patients were followed up from the time of HCCC diagnosis to the time of death or study completion (January 2023).

### IHC

All cases were processed as Formalin-Fixed and Paraffin-Embedded (FFPE) specimens and then sectioned at a thickness of 4.0 μm. Hematoxylin and eosin staining was applied to some sections, while others were used for IHC examination. Immunostaining was performed on the fully automated UltraPath 60 instrument (Beijing Zhongshan Golden Bridge Biotechnology Co., Ltd, Beijing, China). Antigen retrieval was conducted in Tris-based buffer (pH = 9.0) for 20 min using this platform. The endogenous peroxidase was inactivated with 3% hydrogen peroxide for 15 min. Next, the samples were incubated with primary antibodies for 30 min and then secondary antibodies for 20 min. Staining signals were visualized using 3.3'-Diaminobenzidine (DAB). All primary antibodies were purchased from Beijing Zhongshan Golden Bridge Biotechnology Co., Ltd in a ready-to-use format and are listed in Table 1. Ki67, P63, P40, and S-100 staining was localized to the nucleus, while CK7, CK5/6, SMA, GFAP, and calponin staining was localized to the cytoplasm. The proportion of positive tumor cells for Ki67 was scored.For P63, P40, S-100,CK7,CK5/6, SMA, GFAP, and calponin, more than 50% of tumor cells moderately to strongly positive were diagnosed as positive.

**Table 1:** The primary antibodies used in this study.

Antibody	Catalog number	Source	Clone
P63	ZM-0406	Mouse	4A4+UMAB4
S-100	ZM-0224	Mouse	15E2E2 + 4C4.9
GFAP	ZM-0280	Mouse	UMAB129
Ki67	ZM-1066	Mouse	UMAB107
P40	ZM-0472	Mouse	BC28
CK5/6	ZM-0313	Mouse	OTI1C7
CK7	ZM-0071	Mouse	UMBA161
SMA	ZM-0003	Mouse	UMBA237
Calponin	ZM-0176	Mouse	CALP

### FISH

EWSR1 FISH was performed on 4-μm sections obtained from formalin-fixed, paraffin-embedded tissue blocks using an Anbiping (Anbiping Pharmaceutical Technology Co., Ltd; China) EWSR1 break-apart probe. This assay contained a red 439-kb DNA probe and a green 929-kb DNA probe, which flank the 5′ and 3′ ends of the EWSR1 gene on chromosome 22, respectively. Based on our FISH assay validation experiments, EWSR1 fusion positivity was defined as the splitting of the red and green probe signals to a distance larger than the length of the two probes, or as isolated red or green signals in addition to the normal red-green unsplit pair in at least 15% of the scored tumor nuclei.

## Results

### Clinical presentation

The median age of the HCCC patients was 60 (range 28–84) years. The ratio of male to female participants was 4:5. Among the nine patients, five had HCCC in the nasopharynx, two in the base of the tongue, and two in the oropharynx (Details see Table 2). Patients with tumors located in the oropharynx or the base of the tongue had symptoms such as pharyngeal foreign body sensation, speaking as if holding water in your mouth, a sore throat, and difficulty swallowing. Meanwhile, patients with tumors located in the nasopharynx had symptoms such as nasal congestion, a runny nose, bloody nasal discharge, ear tightness, tinnitus, and hearing loss.

**Table 2:** The clinical data of the nine HCCC patients included in this study.

Case	Gender	Age (Y)	Location	Diagnosis time (Y/M)	MD (cm)	Clinical stages	EWSR1 gene	Treatment	Prognosis
1	F	44	Tougue	22/9	4.5	T3NOMO	Break	Removal of left tongue root pharyngeal wall mass + lymph node dissection in cervical areas I‒V	Loss to follow-up
2	M	32	Oropharynx	22/12	4.4	T3NOMO	Break	Endoscopic resection of pharyngeal mass.	No recurrence, no metastasis
3	M	60	Nasopharynx	23/9	4	T1NOMO	Break	Oral nasopharyngeal tumor resection surgery	No recurrence, no metastasis
4	M	64	Nasopharynx	24/1	1.4	T1NOMO	Break	Endoscopic resection of nasopharyngeal mass	No recurrence, no metastasis
5	F	66	Oropharynx	22/12	7.6	T3NOM0	Break	Enlarged resection of a huge mass in the left soft palate and pharyngeal area	Lymph node metastasis after 16-months
6	M	28	Nasopharynx	22/1	3	T1NOMO	Break	Resection of left nasopharyngeal pharyngeal mass via oral route	No recurrence, no metastasis
7	F	84	Tongue	23/2	0.6	T1N0M0	Break	Excision of tongue root tumor via cervical outer route	Local recurrence with lymph node metastasis after 9-months
8	F	60	Nasopharynx	23/11	1.7	T1NOM1	Break	TCb regimen chemotherapy for 2 period of treatment, then, performed endoscopic nasopharyngeal tumor resection	No recurrence, no metastasis
9	F	64	Nasopharynx	14/5	2.1	T2NOMO	Break	Oral nasopharyngeal tumor resection surgery	Relapse after 10-years without metastasis

Y, Year; M, Month; F, Female; M, Male; MD, Maximum Diameter; TCb regimen, Paclitaxel liposomes 240 mg, once every other day, carboplatin 600 mg, once every other day, alternate use, one period of treatment 3-weeks.

**Fig. 1:**
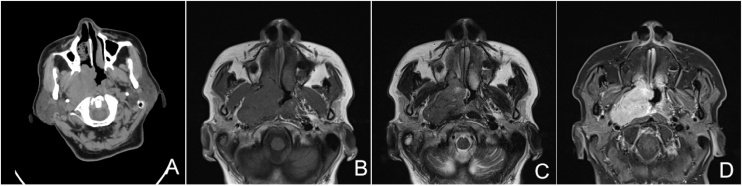
**Clinical imaging evaluation of HCCC.** Computer Tomography (CT) shows expansive masses with unclear boundaries, infiltrating into surrounding soft and bone tissues on the right posterior wall of the nasopharynx (A). Magnetic Resonance Imaging (MRI) shows a weak T1 signal (B), a strong T2 signal (C), and an obvious enhancement of the fat-suppressed contrast-enhanced T1WI (D).

Computer Tomography (CT) and Magnetic Resonance Imaging (MRI) revealed uniform enhancement of the tumor. The CT images of the tumors often showed expansive masses with unclear boundaries, which infiltrated the surrounding soft and bone tissues. MRI showed a weak T1 signal, a strong T2 signal, and an obvious enhancement in the fat-suppressed contrast-enhanced T1 weighted image (Fig. 1).

**Fig. 2:**
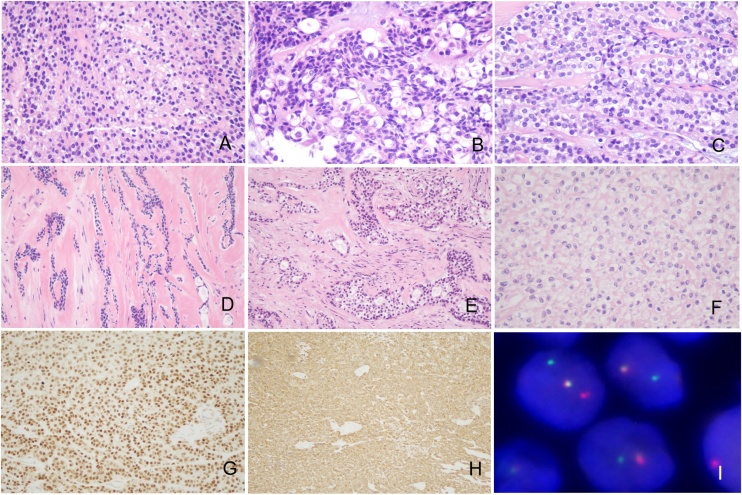
**Hematoxylin and eosin, immunohistochemistry, and fluorescence in situ hybridization evaluation of HCCC tumor sections.** (A) Hematoxylin and eosin staining showed that the HCCC tumors were composed of clear and eosinophilic cells. Tumor cells exhibited mild cellular atypia and were slightly smaller than squamous cells (original magnification ×200). (B) The nuclei of the tumor cells were irregular, with visible nuclear grooves and small nucleoli. The cells had mild morphological abnormalities and lacked intercellular bridges. The transparent tumor cells had a slightly larger cytoplasm than eosinophilic tumor cells. The clear tumor cells resembled plant cells, with a transparent cell membrane and cytoplasm (original magnification ×400). (C–F) The tumor cells were arranged into nests, cords, trabeculae; in some cases, the cells formed small glandular-like structures or were scattered. The tumor cells around the tumor nest were cuboid and arranged in a fence-like pattern, surrounded by characteristic glassy material of varying thickness (original magnification ×200). (G–H) Results of immunohistochemistry analysis, showing positive P63 and CK7 staining (original magnification ×200) (I) Fluorescence in situ hybridization evaluation of EWSR1 rearrangement, the splitting of the red and green probe signals to a distance larger than the length of the two probes, or isolated red or green signals in addition to the normal red-green unsplit pair.

The HCCC tumor was composed of clear and eosinophilic cells. These tumor cells were arranged into nests, cords, and trabeculae. Ducts and small cysts could also be seen. Occasionally, scattered tumor cells were embedded in a densely hyalinized basement-membrane-like material. The presence of varying amounts of hyalinized basement-membrane-like material around the tumor nest was an important feature of HCCC tumors. The tumor cells were small, with wrinkled, and irregular nuclei, visible nuclear grooves, and small nucleoli. The cells displayed signs of mild morphological abnormalities and lacked intercellular bridges. The proportion of clear cells varied within the tumor. The clear cells somewhat resembled plant cells, with a clear membrane and a transparent cytoplasm, which was slightly larger than the cytoplasm of the eosinophilic tumor cells. The tumor cells around the tumor nest were cuboid and arranged in a fence-like pattern. IHC showed positivity for CK7, P63, P40, CK5/6, but negativity for S100, SMA, GFAP, and calponin (Fig. 2). Ki67 index is approximately 5 %–10 %. FISH confirmed EWSR1 rearrangement in all nine patients (Details see Table 2).

### Survival

A patient with HCCC of the oropharynx developed lymph node metastasis at 16 months after surgical resection. A patient with HCCC of the tongue relapsed with lymph node metastasis at 9 months post-resection. One patient with nasopharyngeal HCCC relapsed at 10 years after resection. One patient with HCCC of the tongue was lost to follow-up after surgery. The remaining five patients did not experience recurrence or distant metastasis after surgical resection (Details see Table 2).

## Discussion

HCCC is a rare low-grade malignant tumor of the salivary glands, which is slightly more common in women than in men. Most HCCC tumors develop in the minor salivary glands of the oral cavity, including the soft palate,^4^ the base of the tongue,^5^ nasopharynx,^6^, ^7^, ^8^ lungs,^9^, ^10^ sinuses,^11^ oropharynx,^12^ and occasionally, the lips.^13^ At a cellular level, HCCC tumors are composed of clear and eosinophilic cells, which reside in a variably hyalinized stroma. Despite their name, it is rare for HCCC tumors to be composed entirely of clear cells, and some HCCC tumors lack clear cells altogether. Our evaluation of HCCC tumor morphology revealed that the tumor cells were arranged into nests, cords, and trabeculae. Occasionally, scattered tumor cells were embedded in densely hyalinized basement-membrane-like material. Tumor cells were small, with wrinkled and irregular nuclei, visible nuclear grooves, and small nucleoli. IHC showed positivity for CK7, P63, P40, and CK5/6, but negativity for S100, SMA, GFAP, and calponin. Increased mitotic activity, marked atypia, and necrosis are all indicators of high-grade transformation.^14^, ^15^

A 2011 study by Antonescu et al.,^16^ which used FISH to evaluated 23 patients with salivary gland HCCC, found that the majority of the patients (18/23) harbored the EWSRl-AFT1 fusion gene, further confirming that the most common genetic variation in HCCC was the t(12; 22)(q13; q12) chromosomal rearrangement. Since then, numerous other studies have supported the association between HCCC and EWSR1 rearrangement.^6^, ^10^, ^17^ However, it is worth noting that the EWSRl::AFT1 gene is not unique to HCCC and can also appear in other tumors such as soft tissue clear cell sarcoma and vascular fibrohistiocytoma.^18^

Because of similarities in their morphologies, HCCC is commonly misdiagnosed as squamous cell carcinoma, which is one of the most commonly misdiagnosed tumors in the head and neck region.^19^ The differential diagnosis of HCCC^8^ also includes other salivary gland tumors with clear cells, renal cell-like adenocarcinoma of the nasal cavity and paranasal sinuses, and metastatic renal cell carcinoma. The IHC profiles of HCCC and squamous cell carcinoma are very similar. Generally, the cellular atypia of squamous cell carcinoma is more pronounced than that of HCCC; however, a careful examination of intercellular bridges, keratinization, and in situ cancer areas can help differentiate between the two conditions. Molecular genetic methods such as FISH (e.g., to assess for the presence of the EWSR1-ATF1 fusion gene) can also help clarify the diagnosis. Other salivary gland tumors with clear cells include mucoepidermoid carcinoma, myoepithelial carcinoma, and epithelial-myoepithelial carcinoma. Clear cell mucoepidermoid carcinoma is a rare, aggressive variant of mucoepidermoid carcinoma, which, like HCCC, can also develop in the nasal cavity. Morphological examination and IHC often fail to distinguish between clear cell mucoepidermoid carcinoma and HCCC. However, mucoepidermoid carcinoma is often characterized by the fusion of the CRTC1 and MAML2 genes, which is not a feature of HCCC. Moreover, unlike HCCC, clear cell myoepithelial carcinoma or epithelial-myoepithelial carcinoma express myoepithelial markers, such as SMA, S-100, and calponin. It should be noted that because some clear cell myoepithelial cancers harbor EWSR1 gene rearrangements, FISH alone cannot reliably differentiate it from HCCC.^19^ Renal cell-like adenocarcinoma of the nasal cavity and paranasal sinuses is composed of cytoplasmic clear cuboidal cells which diffusely express S100 and SOX10. By contrast, HCCC tumor cells express P63, P40, and CK5/6, and are characterized by EWSR1 rearrangement. Metastatic renal clear cell carcinoma tumor cells are often arranged in nest- or organ-like structures, with a stroma rich in thin-walled blood vessels. Moreover, these tumor cells express renal cancer markers such as PAX8, PAX2, and CD10. Despite these defining characteristics, a physical examination is often needed to exclude a metastatic tumor diagnosis. Clear Cell Odontogenic Carcinoma (CCOC) and HCCC bear remarkable similarities in biological behavior, histology, immunohistochemical staining, and molecular characteristics.^20^, ^21^, ^22^ CCOC predominantly manifests in the mandible and maxilla, while HCCC is mostly found in the minor salivary glands. The potential relationship between these two entities, and whether they represent the same tumor type presenting at different anatomical sites, demands further investigation and validation.

Although HCCC is classified as a low-grade malignancy, some patients may experience local recurrence, and lymph node or lung metastases. Desai et al., who performed a meta-analysis of 97 studies published between 1983 and 2020, found that, among the 202 HCCC patients examined, 13.9% experienced local and distant metastases and had a local tumor recurrence rate of 18.8%. Another study,^23^ which summarized the long-term follow-up data of 155 patients with HCCC, showed that ∼17.7% experienced local recurrence, ∼3.9% experienced local metastasis, ∼3.5% experienced distant metastasis, and ∼3.8% died as a result of HCCC. At present, there is still controversy over whether postoperative radiotherapy is necessary for the extensive resection of HCCC in the head and neck region. However, in cases where complete resection is not possible, the resection margin is positive, or the disease is at an advanced stage, surgery combined with radiotherapy and chemotherapy may represent a better option.^14^

In summary, HCCC is a rare salivary gland tumor, commonly found in the head and neck region. The accurate diagnosis of HCCC relies on meticulous histopathological evaluation and the exclusion of a related differential diagnosis, aided by IHC analysis and molecular genetic testing. At present, extensive local surgical resection and neck lymph node dissection are the preferred treatment methods for HCCC. Moreover, the prognosis of most patients is favorable. Postoperative adjuvant radiotherapy can be considered based on the outcome of surgical resection to reduce local or regional recurrence.

## ORCID ID

Donglin Ma: 0009-0000-5922-2941

Yahui Li: 0009-0007-2442-2303

Dongmei Yang: 0009-0002-0542-2280

## Declaration of competing interest

The authors declared that they do not have any commercial or associative interest that represents a conflict of interest in connection with the work submitted.

The authors declare no conflicts of interest relating to this work.
